# Implications of p53 protein upregulation in oral lichen planus: a systematic review and meta-analysis

**DOI:** 10.4317/medoral.26808

**Published:** 2024-10-13

**Authors:** Carmen Keim-del Pino, Pablo Ramos-García, Liliana Aparecida Pimenta-Barros, Miguel Ángel González-Moles

**Affiliations:** 1 School of Dentistry, University of Granada, Granada, Spain; 2 Biosanitary Research Institute ibs.GRANADA, Granada, Spain; 3 Federal University of Espirito Santo, Dental Sciences Graduate Program, Vitoria, Brazil

## Abstract

**Background:**

This systematic review and meta-analysis qualitatively and quantitatively analyzes the current evidence on the implications of p53 upregulation in oral lichen planus (OLP) assessed by immunohistochemical techniques, in order to identify molecular mechanisms involved in the behavior of OLP as an oral potentially malignant disorder.

**Material and Methods:**

We searched MEDLINE/PubMed, Embase, Web of Science and Scopus for studies published before February-2024. We critically assessed the methodological quality of primary-level studies and performed meta-analyses.

**Results:**

Twenty-four individual studies met the inclusion criteria, comprising 721 OLP samples, in which the expression of p53 was analyzed through immunohistochemistry. Most OLP displayed p53 protein upregulation (pooled proportion [PP]= 66.76%, 95%CI=54.84-77.76). Regarding the magnitude of association analysis, oral squamous cell carcinoma (OSCC) cases showed a significantly higher frequency according to p53 expression in comparison to OLP (OR=2.79, 95%CI=1.84-4.24; *p*<0.001); while, OLP exhibited a significantly higher frequency for p53 expression in comparison to healthy controls (OR=5.70, 95%CI=2.90-11.19; *p*<0.001).

**Conclusions:**

In conclusion, the present study demonstrates the frequent p53 protein upregulation in patients with OLP, which is probably indicating an antitumor response in an epithelium whose cells are under cellular stress and at risk of cancer.

** Key words:**p53, oral lichen planus, oral potentially malignant disorders, malignant transformation, oral cancer, systematic review, meta-analysis.

## Introduction

Lichen planus is a very prevalent mucocutaneous disease that primarily affects the oral mucosa (OLP) and occasionally the skin, nails, scalp and other mucous membranes. It is a disease of unknown aetiology, of autoimmune nature, in which T-lymphocyte aggression is directed towards the basal and parabasal layers of the oral epithelium and epidermis (1-4). The most relevant fact in OLP is its current consideration as an oral potentially malignant disorder (OPMD) (5), for which has been demonstrated on the basis of evidence a malignancy rate higher than 2% of cases (6-14). It has also been recently reported that oral carcinomas developed from OLP present a significantly better prognosis than conventional oral carcinomas, with better survival rates, smaller size and less probability of lymph node involvement at diagnosis, which seems to be related to inherent features of the own biopathology of the tumour (15). The current consideration of OLP as an autoimmune disease has raised the hypothesis that its malignant transformation may be mediated by the inflammatory infiltrate, which consistently appears in the lesions, as it occurs in other autoimmune diseases (16-20). However, little is known about the molecular mechanisms operating during the malignant transformation of OLP. A research line developed by our group in recent years, applying immunohistochemistry techniques applied to our case series, has reported that essentially a hyperproliferative state develops in OLP with overexpression of ki-67, as well as upregulation of p53 and Bcl-2, and downregulation of caspase-3 and Bax (21-28). We have been asking ourselves why an epithelium that is intensely and chronically attacked by an autoimmune inflammatory infiltrate, which severely damages the epithelial cells by distorting their architecture (vacuolating degeneration) responds with increased proliferation and a marked lack of apoptosis. All this has been interpreted by us as a molecular mechanism aimed to elude apoptosis (upregulation of Bcl-2) and increase the cell proliferation rate, preventing the affected oral epithelium in lichen planus from succumbing to the autoimmune aggression consequently to the massive development of apoptosis in the damaged cells (downregulation of caspase-3 and Bax), thus avoiding the appearance of erosions, which constitutes the most severe form of the disease. Hypothetically, however, this defensive response could involve paying a high price for the epithelium in the form of an increased risk of cancer development, due to the fact that the hyperproliferative and anti-apoptotic state could generate genomic instability and oncogenic mutations (17,29). Despite the aforementioned, OLP is one of the OPMD with the lower risk of malignancy, which is probably due to the proper functioning of tumour suppressor genes, essentially the TP53 gene. We have shown that p53 in OLP acts essentially by inducing cell cycle arrest for the repair of damaged DNA and not by inducing apoptosis (24,28). We now also know that mutations in the TP53 gene in OLP are not frequent (30) and thus, presumably the adequate function of this tumour suppressor could prevent the malignant transformation of this territory predisposed to the development of cancer; On the contrary, the failure of tumour suppressor mechanisms could drastically favour malignization, and since the histopathological -and molecular- alterations in OLP extend widely through the oral mucosa, it is not surprising that once a first carcinoma has developed, an increased risk of multiple carcinomas may be observed, as occurs in cancerization fields and has also been documented in OLP (15,31). Our hypothesis appears to answer several of the questions that arise regarding the molecular process operating in OLP malignancy, although we must acknowledge that it has limited experimental and evidence-based support. In 2022 our research group published a scoping review on the expression of the hallmarks of cancer in OLP and we reported that those distinctive characteristics of cancer which had been studied most extensively and based on evidence in OLP were tumour-promoting inflammation, the existence of sustained proliferative signalling and the evasion of growth suppressor signals/apoptosis evasion capacity, and in all of them the findings support our hypothesis (17). This paper also demonstrated important evidence gaps, in terms of systematic reviews and meta-analyses, among them concerning the tumour suppressor gene p53, which is relevant due to the fact that it is hypothetically one of the main protectors of OLP malignancy. A knowledge of the precise significance of p53 overexpression in OLP, based on the evidence, could have implications essentially prognostic and for the management of OLP, which could perhaps be translated into clinical practice.

In the present paper the results of the only systematic review and meta-analysis performed to date on the implications of p53 protein upregulation in OLP assessed by immunohistochemical techniques are presented, with the aim to draw conclusions for a more appropriate management of the disease in relation to its behaviour as an OPMD.

## Material and Methods

With the purpose of addressing this systematic review and meta-analysis, MOOSE and PRISMA reporting guidelines (32,33) were followed. Additionally, standard methodological criteria were chosen from Cochrane (34) and Joanna Briggs Institute (University of Adelaide, Australia) (35) in order to comply with an adequate study design.

- Protocol

Firstly, a study protocol was accomplished and presented in a worldwide distinctive database (PROSPERO International Prospective Register of Systematic Reviews; registration codes ID549401 / CRD42024549401) pursuing to minimize the risk of bias in order to enhance the transparency, accuracy and integrity of the present systematic review and meta-analysis. Hence, our protocol was supported by PRISMA-P statement, guaranteeing its strict fulfillment (36).

- Search strategy

We searched MEDLINE (through PubMed), Embase, Web of Science, and Scopus databases for studies published before February-2024. As a means to maximize sensitivity, it was built a search strategy combining databases’ thesaurus (i.e., MeSH and EMTREE) with free terms (Supplement 1). Besides, further records were obtained by handsearching the reference lists of retrieved studies and through Google Scholar. All the records’ references and duplicates’ management and remotion were dealt by Mendeley software (v.1.19.8, Elsevier, Amsterdam, The Netherlands).

- Eligibility criteria

The inclusion criteria used were: 1) Original studies, with no restraint by date or publication language; 2) Observational study design; 3) Studies analyzing the differential expression of p53, evaluated through immunohistochemistry in samples from patients presenting OLP, compared or not with healthy mucosa (control group) or oral squamous cell carcinoma (comparison group) samples; and (4) Patients of any age, sex or geographic area.

Exclusion criteria: 1) Studies that do not involve p53 expression, or evaluated by methods other than immunohistochemical technique, in patients presenting OLP; 2) Lichen planus lesions on different anatomical locations or with no distinction among oral, cutaneous or genital lichen planus; 3) Lack of essential statistical data for meta-analyses; 4) Retracted articles, basic research with animals or *in vitro*, secondary/tertiary-level studies (e.g, scoping, systematic or umbrella reviews, with or without meta-analyses), case reports, meeting abstracts, editorials, book chapters, letters, medical hypothesis or personal comments.

- Study selection process

Eligibility criteria were applied individually by two authors (CKDP and PRG). The selection of the articles was achieved by the evaluators in two phases, primarily in the screening by titles and abstracts to include records that seemed to adhere to the inclusion criteria; secondly, the previously selected articles were read and assessed full-text being excluded those that failed to meet the above-mentioned inclusion criteria. Any discrepancies were sorted out through consensus.

- Data extraction

One author (CKDP) extracted information from the selected studies standardized in an Excel spreadsheet data collection form (v.16.53, Microsoft, Redmond, WA, USA). The gathered data comprised information on the first author, year of publication, sample size, language and publication date, country, continent, anatomical subsites, clinical type, sex and age of patients, tobacco, areca nut and alcohol consumption, study design, immunohistochemical methods (i.e., antibody, dilution, incubation time, and temperature), cut-off point for positivity cases, cellular pattern and, regarding p53 expression, the number of positive and negative cases with their respective proportions in the different layers of epithelium, lamina propria and inflammatory infiltrate. Furthermore, number of total and positive p53 cases in healthy controls and OSCC were also collected.

- Appraisal of quality and risk of bias

One author (CKDP) critically valued the methodological quality and risk of bias (RoB) of the primary-level records by Joanna Briggs Institute tool (35,37), designed especially for meta-analyses of proportions: (a) “Was the sample representative of the target population?”; (b) “Were the study participants recruited in an appropriate way?”; (c) “Was the sample size adequate?”; (d) “Were the study subjects and the settings described in detail?”; (e) “Was the data analysis conducted with sufficient coverage of the identified sample?”; (f) “Were objectives, standard criteria used for the measurement of the condition?”; (g) “Was the condition measured reliably?; (h) Was the statistical analysis appropriate?”; (i) “Were all important confounding factors/subgroups/differences identified and accounted for?”; and (j) “Were subpopulations identified using objective criteria?”. The potential risk of bias was qualified as high RoB, unclear/moderate RoB, or low RoB.

- Statistical analysis

To estimate the differential expression of p53 in OLP samples, pooled proportions (PP) were computed with their corresponding 95% confidence intervals (CI). The calculation of these proportions was carried out by the extraction of raw numerators (number of cases with p53 positive expression) and raw denominators (total number of OLP samples). Therefore, 95% CI were built for each individual study by applying the Wilson score method (38). In order to decrease the influence of studies with extreme values (values 0, 100 or close to these), Freeman-Tukey double arcsine transformation was implemented by stabilizing the variance of the specific proportions of each study (39). The resulting transformed PP -expressed as percentages- obtained through meta-analytical methods were subsequently backtransformed. The magnitude of association between the expression of p53 among different groups (i.e., OSCC vs OLP, OLP vs healthy oral mucosa) was also independently investigated calculating and combining odds ratios (OR) with their corresponding 95% CI. Random-effects models were applied to all meta-analyses, weighed by the inverse variance based on the DerSimonian and Laird method (40), in order to consider different underlying results across potential study subpopulations (e.g., differences innate to the variability of experimental methods, such as different antibodies, immunohistochemical pattern, cut-off points, etc) (41). Forest plots were constructed to graphically illustrate the global effect sizes and consequently for visual inspection analyses. Pursuing the assessment of heterogeneity between studies, we utilized Cochran's Q test, based on Chi-square test being *p* < 0.10 presumed as significant due to its low statistical power. In addition, Higgins’ I2 was run to quantified the proportion of heterogeneity in order to rate the proportion of variability in observed effects mirrors variation in true effects, rather than sampling error (42,43). The appraisal of potential sources of heterogeneity were carried out by stratified meta-analyses, also to determine subgroups-specific relative frequencies (44). Moreover, secondary analyses were adopted to verify stability (i.e., “leave-one-out” sensitivity analysis) and reliability of (i.e., small-study effects analysis) meta-analysis results. In an attempt to evaluate small-study effects -for instance publication bias- funnel plots were constructed and the Egger regression test (45) was also applied (considering a pEgger-value < 0.10 as significant). All statistical analyses were executed with Stata software (version 16.1, Stata Corp, USA).

## Results

- Results of the literature search

**Figure 1:**
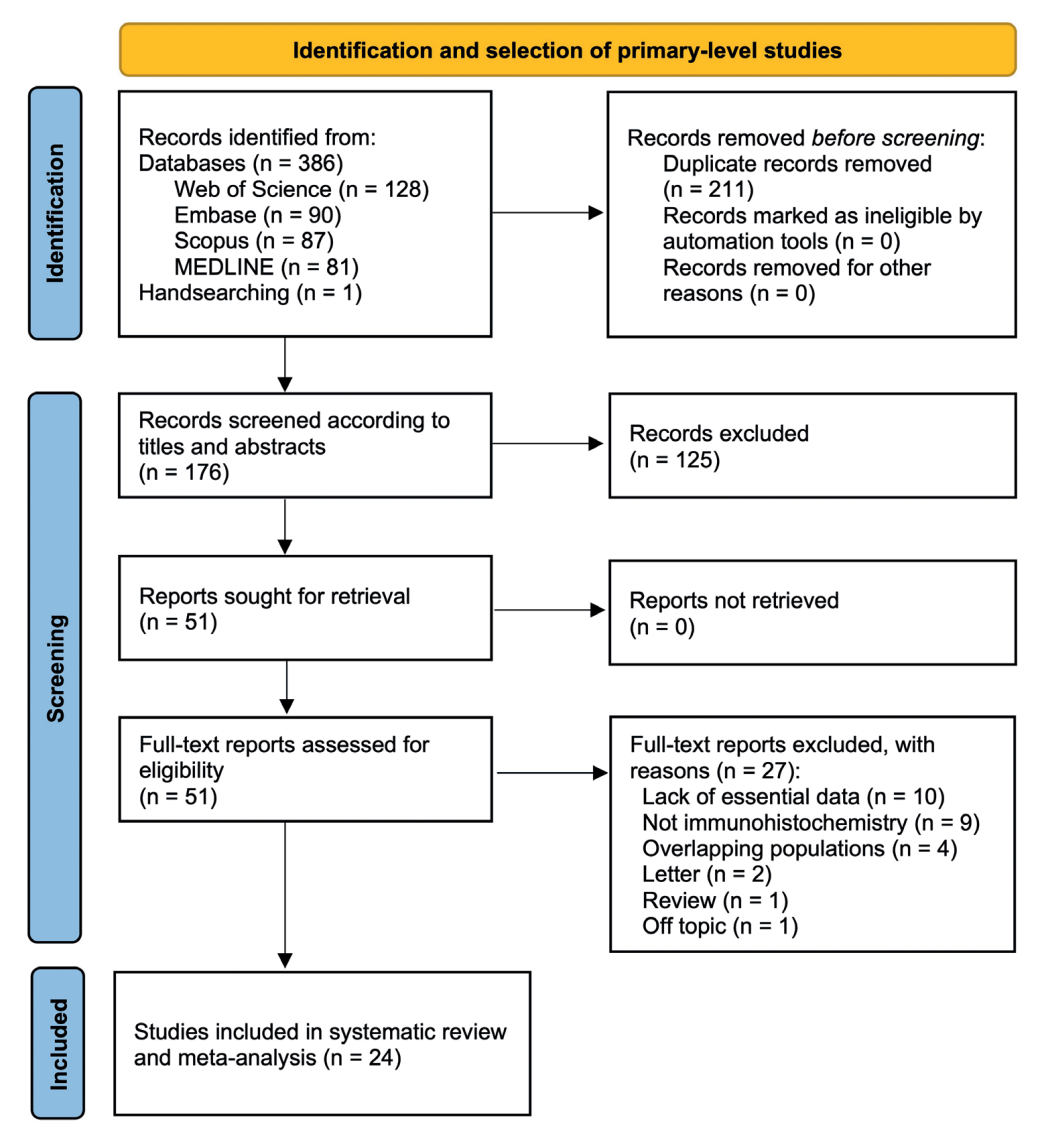
Flow diagram of the process of identification and selection of primary-level studies.

Results derived from the study selection process were captured in the flow diagram (Fig. 1).

A total of 386 records published before February-2024 were retrieved: 128 from Web of Science, 90 from Embase, 87 from Scopus, 81 from Medline/PubMed, and one through handsearching methods. Hereafter duplicates remotion, 176 studies were considered to be potentially eligible. Upon being screened according to titles and abstracts, 51 records were evaluated by full-text reading, of which 27 studies failed to meet the inclusion criteria. At the end, 24 studies (28,46-68) were included in the qualitative and quantitative analysis (all included and excluded studies’ references—with their reasons for exclusion—are listed in the Supplement 2 and Supplement 3, respectively).

- Study characteristics

Table 1 summarizes the general characteristics of the study sample, integrated by 24 primary-level studies systematically reviewed, which comprehended 721 OLP cases (range = 8 - 65 cases), in which the differential expression of p53 was assessed by immunohistochemical technique across retrospective cohorts. Considering the study countries and continents, 8 studies (5 countries) were performed in Asia, 8 studies (6 countries) in Europe, 5 studies (3 countries) in South America, 1 study (1 country) in North America, and also only one study from 1 country was included from Oceania. Supplement 4 exhibits in detail the characteristics and parameters of the study sample.

- Qualitative evaluation

**Figure 2:**
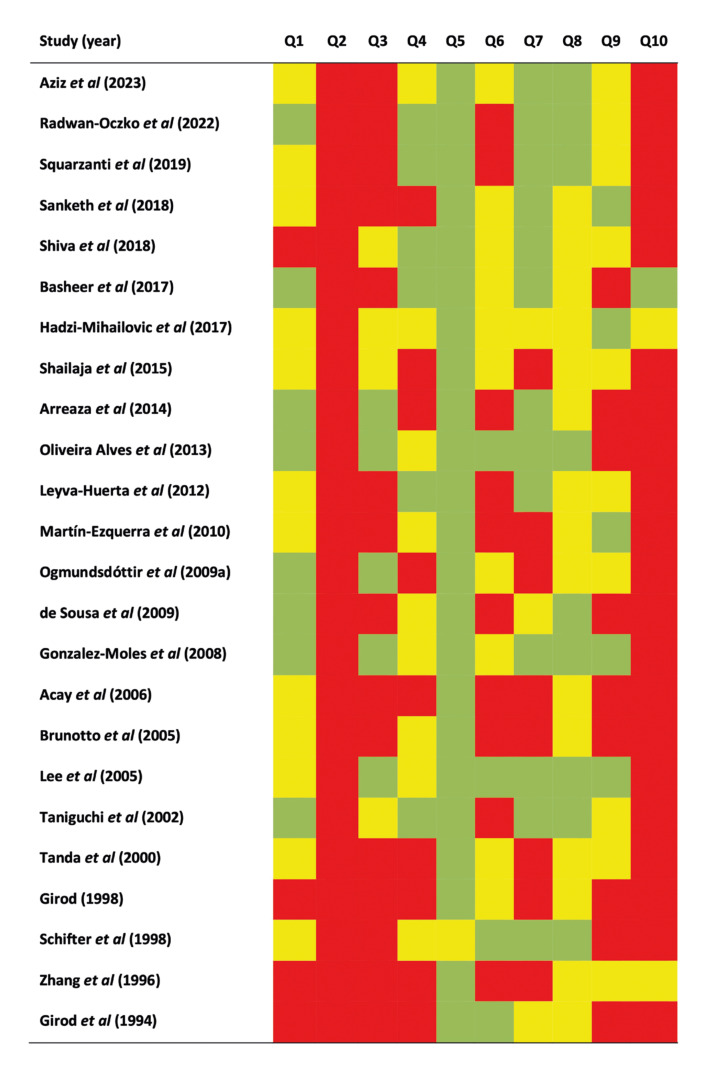
Quality plot graphically representing the risk of bias across primary-level studies, critically appraising ten domains, using a method specifically designed for meta-analyses of proportions (developed by the Joanna Briggs Institute, University of Adelaide, South Australia). Green, low risk of potential bias; yellow, moderate; red, high.

After the methodological quality and risk of bias analysis was performed across primary- level studies, it was determined that all studies were not conducted with the same rigor in accordance with the Joanna Briggs Institute tool. As it was foreseen, the highest risk of potential bias was displayed by items Q2, Q9 and Q10 (Fig. 2).

Regarding domain Q2, there was a lack of application of sampling methods in most of the primary-level studies (i.e., random recruitment methods, statistical calculation of sample size). Concerning domain Q9, primary-level studies failed to communicate potentially confounding variables. (i.e., alcohol or tobacco consumption) and ultimately, domain Q10 depicted insufficient subgroups’ identification using objective criteria (i.e., sex, age, alcohol or tobacco consumption).

- Quantitative evaluation

**Figure 3:**
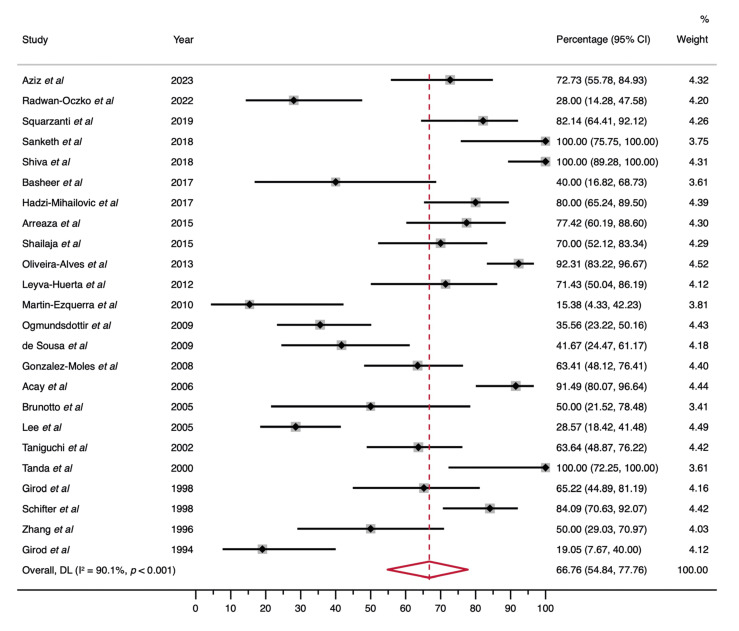
Forest plot graphically representing the differential expression of p53 -using pooled proportions as ES metric, expressed as percentage- among OLP patients. ES, effect size; CI, confidence interval; Random-effects model.

Meta-analysis of proportions. The differential expression of p53 in OLP estimated as pooled proportion (PP) was 66.76% (95% CI = 54.84 - 77.76), with a considerable heterogeneity degree (I2 = 90.1%, *p* < 0.001) (Fig. 3, Table 2). In the stratified analyses, several subgroups preserved p53 overexpression in OLP patients and some of them showed even a higher expression (Table 2, Supplement 5-12).

Meta-analysis on the magnitude of association. Concerning the magnitude of association, OSCC cases showed a significantly higher frequency according to p53 expression in comparison to OLP mucosa samples (OR = 2.79, 95% CI = 1.84 - 4.24; *p* < 0.001) and, lastly, the magnitude of association between OLP and healthy controls demonstrated a significantly higher frequency for p53 in the first group than in the second group (OR=5.70, 95%CI=2.90-11.19; *p*<0.001) (Table 2, Supplement 13, Suppement 14).

Analysis of small-study effects. Visual inspection analysis of the asymmetry of the funnel plots (Supplement 15) and the statistical tests performed for the same purpose confirm the absence of small-study effects on the meta-analyses of the differential expression of p53 in OLP [ pEgger = 0.49], for which biases -e.g., publication bias- could be potentially ruled out. Therefore, our meta-analytic results are reliable from a statistical and epidemiological point of view.

Sensitivity analysis. The sequential repetition of the meta-analysis by performing the so-called “leave-one-out” method (Supplement 16) did not influence on the overall resultant PP of the differential expression of p53 in OLP. Therefore, the reported pooled estimations are sTable.

## Discussion

The results of our systematic review and meta-analysis of 24 studies and 721 patients regarding the upregulation of p53 protein in OLP lesions show that 66.76% of cases show increased expression of this tumour suppressor (95%CI=54.84-77.76). This is an important observation as it indicates that cellular stressors are occurring in OLP, probably linked to DNA damage associated with autoimmune aggression, which triggers the TP53 gene out of its "standby" state, activating post-translational mechanisms that increase p53 protein production and its stabilisation in the nucleus to exert its tumour suppressor functions (69). Currently, we know that TP53 gene mutations are very infrequent in OLP (30), which seems to indicate that the overexpression of the protein is essentially due to its wild-type form which, in this scenario, presumably exerts mechanisms of cell cycle arrest and DNA damage repair and, much less frequently, mechanisms linked to the development of apoptosis. This idea is supported by the scarcity of apoptosis markers that are usually expressed in OLP, despite the intense cellular damage linked to the autoimmune aggression that appears in this disease (22,24-26). We are aware that immunohistochemical overexpression of p53 protein is not an equivalent marker of gene mutational status, and discrepancies between immunohistochemical data and TP53 sequencing results have been demonstrated up to 40% of cases (70). However, there is general agreement that overexpression of p53 and its accumulation in the nucleus indicate that something is wrong in the cell, which is also demonstrated by an interesting result of our meta-analysis: while oral squamous cell carcinoma shows p53 overexpression 2.79-fold higher than in OLP, the overexpression of this protein is 5.70-fold higher in OLP vs. normal oral mucosa, i.e. in relation to p53, OLP closely resembles cancer than normal oral mucosa. There are no primary-level studies analysing how p53 overexpression behaves in cases of OLP progressing to cancer, and this is a research line that should be established in the future. We do know, however, that immunohistochemical overexpression of p53 behaves as a risk marker for progression to cancer in other OPMD, essentially in oral leukoplakia (71), which suggests that this could probably also occur in OLP. In any case, as we have discussed, p53 upregulation in OLP is an indicator of the fact that epithelial cells are in a dangerous molecular situation and under risk of malignant transformation. In our opinion, p53 behaves in lichen planus as a guarantee of persistence in benignity (17). However, we must recognise that since the epithelium of OLP is essentially hyperproliferative and subject to cell survival mechanisms, and under constant immune aggression, the role of p53 as tumour suppressor is likely to be very hard; thus, its failure, for example due to mutational events in the gene, will presumably be a determining factor in the malignancy of the disease. TP53 mutations are relevant in oral cavity cancer, ranging from 38% to 64% according to case series (29,72-74), and in the head and neck region a common mechanism of TP53 mutations is associated with tobacco use. In this regard, our research group has recently shown that tobacco use is one of the main risk factors for the progression to cancer in patients with OLP (6), which is probably linked to the loss of the relevant roles played by this gene in OLP. It could therefore be suggested that tobacco consumption could act as a co-factor of the inflammatory infiltrate in the malignant transformation process of OLP, although we do not know whether the effects are additive or multiplicative. In any case, it is imperative that OLP smokers stop the habit.

Our study has also shown that there are no significant differences in p53 upregulation in different geographic areas, specifically in the comparison of Asian cases to the rest of the world (77.21% vs. 61.62%, respectively; *p* = 0.26). This is relevant due to the fact that in some Southeast Asian countries large amounts of tobacco are consumed and consequently, our finding indicates that the upregulation of p53 in OLP is essentially due to mechanisms inherent to the disease process and not to other reasons

According to our qualitative evaluation using Joanna Briggs Institute tool, we also should point out that the studies included in this systematic review and meta-analysis have not been conducted with the same methodological rigor, most of them presenting a high risk of potential bias across three specific domains related to sampling method, potentially confounding factors and the failure to report relevant several clinic-demographical variables (e.g., sex and age distributions, or tobacco consumption). This is actually an inherent limitation of the methodological design of primary-level studies published of this topic. Nevertheless, after applying a stratified meta-analysis to assess the influence of risk of bias on the overall results, we did not find significant differences between subgroups, which means that the methodological quality of the primary-level studies did not influence on the differential expression of p53 in OLP and, consequently, on the results of this meta-analysis. Anyway, the recommendations and potential biases reported through this systematic review should be followed in future primary-level studies in order to improve and standardize future research.

## Conclusions

In conclusion, the present meta-analysis demonstrates a frequent overexpression of p53 protein in OLP that probably indicates an antitumor response in an epithelium whose cells are under cellular stress and at risk of cancer. We do not know how the expression of this protein behaves in cases of OLP progressing to cancer, and this is a future line to be implemented; however, in our opinion, p53 upregulation should be considered as, first, an additional evidence of the premalignant character of OLP and, second, as an alarm signal for the clinician.

## Figures and Tables

**Table 1:** Summarized characteristics of the study sample.

Total		24 studies
Year of publication		1994-2023
Total patients (range)		721 (8-65)
Study design	Retrospective cohort	24 studies
P53 immunohistochemical pattern	Nuclear staining	16 studies
	Mixed staining	2 studies
	Not reported	6 studies
Anti-p53 antibody	DO7	19 studies
	1801	2 studies
	Ab-5	1 study
	Not reported	2 studies
Cut-off point for p53 overexpression	1%	8 studies
	5%	10 studies
	Not reported	6 studies
Anti-p53 antibody dilution	1:50	3 studies
	1:100	4 studies
	1:200	3 studies
	Not reported	14 studies
Anti-p53 antibody temperature of incubation	4ºC	8 studies
	Room Temperature	4 studies
	Not reported	12 studies
Anti-p53 antibody time of incubation	≤60 minutes	8 studies
	Overnight	8 studies
	Not reported	8 studies
Geographical region	Asia	8 studies, 5 countries: India, Iran, Iraq, Japan, Taiwan
	Central America	1 study, 1 country: Mexico
	Europe	8 studies, 6 countries: Germany, Iceland, Italy, Poland, Serbia, Spain
	North America	1 study, 1 country: Canada
	Oceania	1 study, 1 country: Australia
	South America	5 studies, 3 countries: Argentina, Brazil, Venezuela
Total		6 continents (17 countries)

**Table 2:** Meta-analyses on the differential expression of p53 in oral lichen planus samples.

Meta-analyses		No. of studies	No. of patients	Stat. Model	Wt	Pooled data		Heterogeneity	
						ES (95% CI)	*P-value*	*P_het_*	*I^2 ^*(%)
Differential p53 expression in OLP^a^		24	721	REM	D-L	PP=66.76% (54.84-77.76)	──	<0.001	90.1
Subgroup analysis by geographical region^b^	Asia	8	227	REM	D-L	PP=77.21% (52.95-94.97)	0.26^c^	<0.001	92.5
	Non-Asia	16	494	REM	D-L	PP=61.62% (47.67-74.73)		<0.001	89.1
Subgroup analysis by immunohistochemical pattern^b^	Nuclear	16	488	REM	D-L	PP=71.89% (58.19-83.93)	0.07^c^	<0.001	89.3
	Mixed	2	77	REM	D-L	PP=76.91% (4.40-100.00)		<0.001	98.1
	Not reported	6	156	REM	D-L	PP=48.73% (34.06-63.49)		0.009	67.2
Subgroup analysis by anti-p53 antibody^b^	DO-7	19	603	REM	D-L	PP=63.86% (49.69-77.05)	0.25^c^	<0.001	91.6
	Other	3	77	REM	D-L	PP=84.66% (63.62-98.25)		0.03	72.0
	Not reported	2	41	REM	D-L	PP=67.14% (45.60-85.81)		0.23	29.4
Subgroup analysis by cutoff point for p53 overexpression^b^	0%	7	228	REM	D-L	PP=85.97% (73.98-94.97)	0.01^c^	<0.001	79.1
	5%	10	326	REM	D-L	PP=57.37% (39.94-73.96)		<0.001	89.2
	Not reported	7	167	REM	D-L	PP=56.58% (27.76-83.38)		<0.001	92.1
Subgroup analysis by anti-p53 antibody dilution^b^	1:50	3	108	REM	D-L	PP=91.06% (77.03-99.37)	0.002^c^	0.05	66.7
	1:100	4	129	REM	D-L	PP=39.23% (18.04-62.56)		0.001	82.7
	1:200	3	91	REM	D-L	PP=65.36% (38.29-88.19)		0.002	84.4
	Not reported	14	393	REM	D-L	PP=68.05% (52.48-81.94)		<0.001	89.3
Subgroup analysis by antibody temperature of incubation^b^	4ºC	8	263	REM	D-L	PP=49.59% (27.16-72.10)	0.02^c^	<0.001	91.2
	Room temperature	4	117	REM	D-L	PP=84.38% (72.59-93.59)		0.01	52.2
	Not reported	12	341	REM	D-L	PP=70.75% (54.25-85.04)		<0.001	89.2
Subgroup analysis by antibody time of incubation^b^	≤60 minutes	8	264	REM	D-L	PP=85.57 (74.67-94.03)	0.001^c^	<0.001	77.8
	Overnight	8	242	REM	D-L	PP=47.81 (29.76-66.13)		<0.001	86.9
	Not reported	8	215	REM	D-L	PP=63.11 (40.86-82.99)		<0.001	89.6
Subgroup analysis by overall risk of bias in primary-level studies^b^	Low RoB	10	374	REM	D-L	PP=68.74% (49.76-85.08)	0.75^c^	<0.001	90.1
	High RoB	14	347	REM	D-L	PP=65.18% (49.19-79.73)		<0.001	88.0
Magnitude of association^d^	OSCC vs OLP	10	731	REM	D-L	OR=2.79 (1.84-4.24)	<0.001	0.35	9.6
	OLP vs healthy controls	14	593	REM	D-L	OR=5.70 (2.90-11.19)	<0.001	0.12	33.2

Abbreviations: Stat., statistical; Wt, method of weighting; ES, effect size estimation; PP, pooled proportions; OR, odds ratio; CI, confidence intervals; REM, random-effects model; D-L, DerSimonian and Laird method; OLP, oral lichen planus; RoB, risk of bias. a- Meta-analysis of proportions; b- Subgroup meta-analyses; c- Test for between-subgroup differences; d- Meta-analysis of aggregate (summary) data.
